# Q&A with Editorial Board Member Dr Satoshi Honda

**DOI:** 10.1038/s42004-023-00943-0

**Published:** 2023-07-06

## Abstract

Dr Satoshi Honda talks to us about pursuing his scientific dreams, scientific developments he is excited about, directions polymer synthesis and materials development should focus on, as well as his experience of being an Editorial Board Member for *Communications Chemistry*.

Dr Satoshi Honda is an Assistant Professor of Chemistry at the University of Tokyo (Japan), where his research focuses on the synthesis of polymers with complex architectures, the construction of functional nanostructures, and the development of stimuli-responsive organic and polymeric materials.

**Figure:**
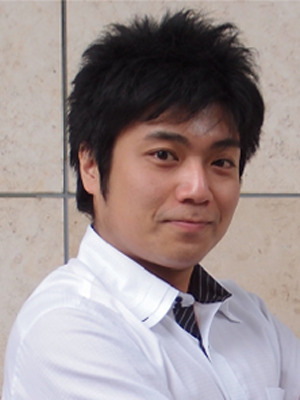
Satoshi Honda

Dr Honda received his PhD from Tokyo Institute of Technology in 2013 for his work on the synthesis and self-assembly of cyclic polymers with Professor Yasuyuki Tezuka. Following his PhD, he spent two years at the Tokyo University of Science as Specially Appointed Assistant Professor. He joined the Department of Basic Science at the University of Tokyo as Assistant Professor in 2015 and was named an Excellent Young Researcher in 2018. During his current position, he engaged in ring-opening-polymerization of unusual cyclic molecules with organic catalysts with Professor Robert M. Waymouth at Stanford University as a Visiting Scholar in 2018-2019. He is the recipient of the Young Scientists’ Prize, Commendation for Science and Technology by Japanese Minister of Education, Culture, Sports, Science and Technology, 2020.

Why did you choose to be a scientist?

Scientists are those who can pursue dreams based on science. I am interested in using the power of material science to realize the imaginary matters of sci-fi movies. No one but a chemist can do this, so I want to enjoy it to my heart’s content.

What scientific development are you currently most excited about?

Technologies that connect materials science with the immersive experience in virtual and augmented reality, i.e., cross reality, in a sustainable future are at the top of my list. I am also interested in the future development of organic and polymerization reactions and materials development using artificial intelligence. There is a limit to what one person can learn. However, I’m thrilled to see how the fusion of AI learning from vast amounts of data and the unusual ideas of unique and talented chemists will open-up the future.

What direction do you think your research field should go in?

I hope to see an increase in the number of pioneering developments that will create new research through deeper involvement between the field of advanced small molecules and polymer synthesis and device development. I think one area that is creating a virtuous circle is 3D printing. In particular, the performance of vat-polymerization (VP) 3D printing (3DP) is progressing in synergy with the development of high-resolution liquid-crystal displays (LCDs). That is, inexpensive 3D printers used for VP 3DP can employ an LCD we’d normally use as a monitor to control the photocuring area. The photoresin cures only in the area where the image is projected on the LCD. As the resolution of LCDs improves, it becomes possible to produce high-quality and intricate 3D models, and new applications can be found for them, such as flow reactors and microfluidic devices. In addition, the development of materials that can be applied to VP-3D is progressing spectacularly.

Such progress in the development of devices and materials should occur in my research field as well, and I would like to do my best to play a part in it.

What attracted you to becoming an Editorial Board Member for *Communications Chemistry*?

When we work as researchers, we have opportunities not only to publish our own research but also to review manuscripts from other researchers. Of course, these are our responsibilities to contribute to science, but there is one more entity that we should not forget, that is, editors of journals. Editors contribute to scientific research in ways different from authors or reviewers with a great deal of responsibility. The editors at *Communications Chemistry* are, of course, responsible for making the journal attractive. In addition, editorial activities at *Communications Chemistry* allow me to think about how to make our research fields more attractive to the readers of the journal while also looking at what is going on in other fields. The opportunity to think about the features and attractiveness of a particular field among a variety of fields will eventually work positively for my own research.

Editorial Board Members should contribute to the development of all areas of chemistry from a more bird’s eye view. I decided to become one of the Editorial Board Members (EBMs) of *Communications Chemistry* because this journal covers all aspects of chemistry, and I expected to learn much from communication with in-house editors and other EBMs. For a more personal reason, being offered the EBM position directly by the Chief Editor was more than enough to move me to take action. Therefore, the moment I received the offer, I immediately decided to accept it.

What have you gotten out of the experience of being an Editorial Board Member for *Communications Chemistry*?

Being an EBM has allowed me to see different views that I would not be able to see if I only served as an author or reviewer. What do editors think about the papers they handle? What do they consider key points and trivial? We might not be able to understand these perspectives until experiencing editorial work ourselves. I strongly feel that this will be essentially the same for other journals, and in turn, will be a great benefit to my own research.

What do you see as the role of *Communications Chemistry* in the scientific community?

At *Communications Chemistry*, decisions at any step are made based on close communication between in-house editors and EBMs. I feel that this interaction ensures the quality of the papers and reduces the blurring of quality. I believe that the role of *Communications Chemistry* is to continue this style of communication and continue to provide high-quality papers in an open access manner. I also think *Communications Chemistry* is building a good community among authors, reviewers, EBMs, and in-house editors through our Collections, the ongoing Reviewer of the Month program, a past Meet the Editors initiative, and various other projects. I am sure that authors, reviewers, EBMs, and in-house editors will continue to build good relationships and will come up with various projects that will ignite the chemistry community. Stay tuned!

*This interview was conducted by the editors of Communications Chemistry*.

